# Diagnostic value of triglyceride and cystatin C ratio in diabetic kidney disease: a retrospective and prospective cohort study based on renal biopsy

**DOI:** 10.1186/s12882-022-02888-3

**Published:** 2022-07-27

**Authors:** Jing Wei, Bo Wang, Feng-jie Shen, Ting-ting Zhang, Zan Duan, Dong-mei Zhou

**Affiliations:** 1 Department of Endocrinology, The Affiliated Hospital of Xuzhou Medical University, Xuzhou, 221000 People’s Republic of China; 2 Department of Endocrinology, The First People’s Hospital of Xuzhou, Xuzhou, 221000 People’s Republic of China; 3 Department of Pathology, Xuzhou Cancer Hospital, Xuzhou, 221000 People’s Republic of China; 4 Department of Rheumatology and Immunology, The Affiliated Hospital of Xuzhou Medical University, Xuzhou, 221000 People’s Republic of China

**Keywords:** Triglyceride and cystatin C ratio, Diabetic kidney disease, Diagnosis, Renal biopsy

## Abstract

**Background:**

Currently, there is a lack of clinical indicators that can accurately distinguish diabetic kidney disease (DKD) from non-diabetic kidney disease (NDKD) in type 2 diabetes. The purpose of this study was to investigate the diagnostic value of triglyceride and cystatin C (TG/ Cys-C) ratio in DKD. Nowadays, there are few studies on the differential diagnosis of TG/ Cys-C ratio between DKD and NDKD.

**Methods:**

The clinical data of patients with type 2 diabetes complicated with proteinuria who underwent renal biopsy from January 2013 to September 2019 in 2 hospitals in Xuzhou were retrospectively collected. According to the pathological classification of kidney, 25 patients in group DKD and 34 patients in non-diabetic kidney disease (NDKD) group were divided into two groups. The admission information and blood biochemical indexes of all patients with renal biopsy were collected, and the TG / Cys-C ratio was calculated. Logistic regression analysis was used to analyze the related factors of DKD in patients with type 2 diabetes and proteinuria. Receiver operating characteristic (ROC) curve was used to evaluate the diagnostic value of TG/Cys-C ratio for DKD in patients with type 2 diabetes and proteinuria. Another 37 patients with type 2 diabetes complicated by proteinuria who were treated in the Department of Nephrology, four hospitals in Xuzhou from October 2019 to October 2021 were selected as the research objects. The TG/Cys-C value cut-off value selected in the retrospective study was selected as the boundary point and divided into two groups according to the values of greater than or equal to the tangent point and less than the tangential point. Serum triglyceride and cystatin C levels were measured and TG / Cys-C ratio was calculated. All patients underwent ultrasound-guided fine-needle renal biopsy. The positive rates of DKD diagnosis in the two groups were compared to verify the predictive value of TG / Cys-C ratio in the diagnosis of DKD.

**Results:**

Retrospective study showed that compared with group NDKD, the DKD group had higher systolic blood pressure, higher cystatin C and creatinine, more diabetic retinopathy, longer duration of diabetes, lower hemoglobin concentration, lower glomerular filtration rate, lower cholesterol, lower triglyceride and lower TG/ Cys-C ratio (*P* < 0.05).Multivariate Logistic regression analysis showed that TG/Cys-C ratio (OR = 0.429, *P* = 0.009) was a protective factor for DKD in patients with type 2 diabetes and proteinuria. Diabetic retinopathy (OR = 7.054, *P* = 0.021) and systolic blood pressure (OR = 1.041, *P* = 0.047) were independent risk factors for DKD in patients with type 2 diabetes complicated with proteinuria. ROC curve showed that the area under the curve predicted by TG/Cys-C ratio for the diagnosis of DKD was 0.816, the sensitivity was 84%, and the specificity was 67.6%. The tangent value of TG / Cys-C ratio is 2.43. Prospective studies showed that in 37 patients with type 2 diabetes and proteinuria, 29 patients had a TG/Cys-C ratio of less than 2.43. The TG/Cys-C ratio of 8 patients was more than 2.43. Ultrasound guided fine needle aspiration biopsy revealed that 22 of the 29 patients had pathological diagnosis of DKD, sensitivity 91.67%, specificity 46.15%, positive predictive value 75.80%, and negative predictive value 75%.

**Conclusion:**

In type 2 diabetic patients with proteinuria, the ratio of TG/Cys-C has certain predictive value for the diagnosis of DKD.

## Introduction

Diabetic kidney disease (DKD) is one of the main microvascular complications of diabetes. Estimated Glomerular Filtration Rate (eGFR) and/or elevated Urine albumin-to-creatinine ratio (UACR) are characterized [1]. In the past, the diagnosis of DKD was almost always based on clinical manifestations; however, not all patients with proteinuria or decreased renal function were diagnosed with DKD [2]. It has been reported that the detection rate of non-diabetic kidney disease (NDKD) in type 2 diabetic patients undergoing kidney biopsy is increasing [3, 4]. Kidney biopsy is the gold standard for differentiating DKD from NDKD [5], but it is invasive and not suitable for routine clinical practice. Therefore, it is of great clinical significance to actively seek more sensitive and non-invasive markers for differential diagnosis of DKD and NDKD. Previous studies have suggested that abnormal lipid metabolism and lipid deposition are closely related to the occurrence and development of DKD [6]. It has also been pointed out that cystatin C can be used as a biomarker for early detection of DKD patients and can better reflect renal function changes in DKD patients [7]. Based on the renal pathology of diabetic patients, this study intends to explore the clinical significance of TG/Cys-C ratio in the differential diagnosis of DKD and NDKD, and to verify the value of TG/Cys-C ratio in the differential diagnosis of DKD and NDKD.

## Methods

### Patients and study design

The clinical data of patients with type 2 diabetes complicated with proteinuria who received renal biopsy from January 2013 to September 2019 in two Hospitals of Xuzhou (Xuzhou Medical University Affiliated Hospital and Xuzhou Central Hospital) were retrospectively collected. The inclusion criteria were as follows: a. T2DM was diagnosed according to the 2013 American Diabetes Association (ADA) criteria [8]; b.proteinuria was diagnosed when the urinary albumin to creatinine ratio (UACR) > 30 mg/g; c. renal biopsy (male or female) age > 18 years old with clear pathological results. The exclusion criteria were as follows: a. incomplete data or unclear medical history; b. combined with other acute complications of diabetes mellitus; c. Those who had taken fibrates triglyceride lowering drugs within 3 months before renal biopsy; d. Complicated with serious infection of other systems, failure of important organs, systemic immune system diseases and malignant tumors; e. proteinuria appeared before diagnosis of type 2 diabetes; f. The pathological manifestation was DKD combined with NDKD. According to the pathological results of renal biopsy, they were divided into DKD group (25 cases) and NDKD group (34 cases). This study was approved by the ethics committee of the Affiliated Hospital of Xuzhou Medical University (ethics No.: XYFY2021-KL073-01). All enrolled patients who participated in the biopsy of kidney biopsy signed written informed consent.

From October 2019 to October 2021, 37 patients with type 2 diabetes complicated with proteinuria were selected from the Department of Nephrology, four hospitals in Xuzhou (Affiliated Hospital of Xuzhou Medical University, Xuzhou Central Hospital, Xuzhou First People's Hospital and Xuzhou Traditional Chinese Medicine Hospital). According to the previous retrospective study, the cut-off point value of TG/Cys-C ratio for the diagnosis and prediction of DKD was 2.43. Taking the ratio ≥ 2.43and < 2.43 as the defined values, the subjects were divided into two groups. The TG/Cys-C ratio of 29 patients was less than 2.43, including 20 males and 9 females; The age ranged from 26 to 69 years, with an average of (50.00 ± 11.33) years. TG/Cys-C ratio ≥ 2.43 in 8 patients, 6 males and 2 females; The age ranged from 38 to 70 years, with an average of (53.63 ± 10.25) years. There was no significant difference between the two groups in terms of gender and age, and the two groups were comparable. The inclusion criteria were as follows: a. age ≥ 18 years; b. T2DM was diagnosed according to the 2022 American Diabetes Association (ADA) criteria [9]; c. Urinary albumin to creatinine ratio (UACR) > 30 mg/g; d. Underwent ultrasound-guided renal biopsy. The exclusion criteria were as follows: a. With acute complications such as diabetic ketoacidosis; b. Those who had taken fibrates triglyceride lowering drugs within 3 months before enrollment; c. Patients with severe infection of other systems, failure of important organs, diseases of systemic immune system and malignant tumors; d. Type 2 diabetes mellitus was diagnosed as CKD before diagnosis; e. Those who refuse to participate in the experiment. This study was approved by the ethics committee of the Affiliated Hospital of Xuzhou Medical University (ethics No.: XYFY2019-KL149). All enrolled patients who participated in the biopsy of kidney biopsy signed written informed consent.

### Review research indicators

#### General information was collected

The clinical data of all patients from January 2013 to September 2019 were collected, including age, gender, duration of diabetes (the time from the first diagnosis to the time of renal biopsy), whether diabetic retinopathy was found, whether smoking and drinking history, height, weight, systolic blood pressure and diastolic blood pressure were calculated, and body mass index (BMI) was calculated. The blood test indexes of renal biopsy patients were collected, including hemoglobin, fasting blood glucose, albumin, cholesterol, triglyceride, uric acid, creatinine, glomerular filtration rate (eGFR), high-density lipoprotein-c, low-density lipoprotein-c, Cystatin C, glycosylated hemoglobin, fibrinogen (FIB), routine urine chemistry and urinary sediment quantification, and total 24-h urinary protein.TG/Cys-C ratio was calculated, and eGFR was estimated by kidney disease diet improvement (MDRD) formula [10]: eGFR[ml/(min*1.73m2)] = 30,849 × (Scr)^-1.154 × (age)^-0.203 × (0.742female). The pathological report of renal puncture was provided by Nanjing Jinyu medical laboratory.

#### Experimental data collection

Develop unified and detailed forms, recording the gender, age, duration of diabetes, height and weight, Body Mass Index (BMI), BMI = body weight (kg)/height (m)^2^, measuring seat blood pressure, recording systolic and diastolic blood pressure (mmHg).

All subjects were fasted for 8-10 h, and they should avoid drinking one day in advance and eating high-fat protein diet. 10 ml blood was collected from the median vein of the cubital fossa of the forearm on an empty stomach the next morning (vigorous activities and brisk walking should be avoided 15 min before blood drawing). After standing at room temperature for 30 min, 3 ml was centrifuged at 1000 rpm for 5 min, the lower blood cells were removed, the upper serum was taken, the triglyceride level was detected by GPO-POD colorimetry, the serum cystatin C level was detected by immunoturbidimetric method, the creatinine level was detected by creatine oxidase method, and the glycosylated hemoglobin was detected by high performance liquid chromatography. TG/Cys-C ratio was calculated and eGFR was estimated: it was calculated by kidney disease diet improvement (MDRD) formula [10].

All subjects underwent 24-h quantitative detection of urinary protein: empty the bladder at 8 a.m. on the same day, then count the time, collect all urine until 8 a.m. on the next day in a clean container, accurately measure the total amount of urine, record it, stir it evenly, and take 100 ~ 200 ml for examination. In order to prevent deterioration of urination, preservatives (such as 1 ml of 40% formaldehyde solution) can be added to the bedpan during urine collection. The total amount of urinary protein was detected by turbidimetry.

All subjects underwent fundus photography: fundus photography was performed with VISUCAM S224 fundus camera. Two doctors of deputy director of Ophthalmology and above were evaluated.

All subjects were examined by ultrasound-guided fine needle renal biopsy: the ultrasonic instrument was Philips EPIQ7 ultrasonic diagnostic instrument, and the probe model was C5-1; The puncture needle shall be American automatic bard biopsy gun, 16 g × 16 cm, the length of biopsy tissue strip was 22 mm. The position, size (long × thick × width) and thickness of renal parenchyma of both kidneys were routinely examined before renal puncture, thickness of renal parenchyma, unit: cm; Laboratory examination of liver and kidney function, coagulation function, platelet count, blood pressure, preoperative discontinuation of anticoagulants. Empty bladder, puncture position: lie prone, abdominal pillow or cushion, fix the position, conduct ultrasonic positioning, routine disinfection and towel laying, after 2% lidocaine anesthesia, use 16G puncture needle under the guidance of ultrasound, enter the needle to the front edge of the capsule of the lower pole of the kidney, pull the trigger, and take out the tissue strip for biopsy. Continuous compression puncture for 6–8 h after operation. All pathological examinations were sent to Nanjing Jinyu Medical Laboratory for pathological diagnosis. Light microscope (HE, PAS, PASM, Masson staining), electron microscope and fluorescence immunopathological examination were performed respectively, which were analyzed by two experienced pathologists.

#### Statistical analyses

SPSS 20.0 statistical software was used to analyze the data. Through the normality test, the data conforming to the normal distribution are expressed in, and the independent sample t-test is used for the comparison between groups. Those that do not conform to the normal distribution are represented by M (Q1, Q3), and Mann Whitney U test is used for inter group comparison. The number of cases (%) was used for classification and counting data, and the comparison between groups was used χ2 test or Fisher test. Logistic regression analysis was used to analyze the related factors of DKD in patients with type 2 diabetes and proteinuria. The diagnostic value of TG/Cys-C ratio in patients with DKD was evaluated by receiver operating characteristic (ROC) curve, and the cut-off point value was selected. The difference was statistically significant (*P* < 0.05).

## Results

### Patient characteristics in retrospective study

Compared with group NDKD, the proportion of diabetic retinopathy in group DKD was higher than that in group DKD. Systolic blood pressure, duration of diabetes, Cys-C and creatinine in group DKD were higher than those in group NDKD, and the ratios of hemoglobin, glomerular filtration rate, cholesterol, triglyceride and TG/Cys-C in DKD group were lower than those in group NDKD (*P* < 0.05) (Table 1).

**Table 1:** Comparison of clinical characteristics between the two groups

Clinical parameters	DKD Group(*n* = 25)	NDKD Group(*n* = 34)	Z/t/x^2^	*P* value
Male(number, %)	17(68)	18(53)	1.354	0.245
Age (years,‾x ± s)	48.36 ± 12.26	53.12 ± 10.90	-1.571	0.122
Hypertension(number, %)	19(76)	20(59)	1.897	0.168
Tobacco smoking(number, %)	8(32)	4(12)	3.641	0.056
Alcohol drinking(number, %)	2(8)	5(15)	0.620	0.431
DR(number, %)	14(56)	4(12)	13.297	< 0.001
BMI [kg/m2, M (Q1, Q3)]	24.97(22.54,26.26)	22.86(21.48,27.68)	-0.784	0.433
SBP(mmHg, ‾x ± s)	154.48 ± 22.95	138.56 ± 17.04	3.060	0.003
DBP [mmHg, M(Q1,Q3)]	87(80,100)	80(73,94)	-1.169	0.242
Diabetes duration [month, M (Q1, Q3)]	60(24,132)	12(6,72)	-2.418	0.016
HbA1C [%, M (Q1, Q3)]	7.00(5.75,9.37)	7.10(6.70,7.90)	-0.133	0.894
Hb (g/L, ‾x ± s)	113.40 ± 21.32	135.15 ± 20.34	-3.977	< 0.001
FPG (mmol/L, ‾x ± s)	8.12 ± 3.43	6.80 ± 2.08	1.716	0.095
ALB(g/L,‾x ± s)	34.09 ± 7.63	30.94 ± 9.39	1.379	0.173
TC (mmol/L,‾x ± s)	6.08 ± 1.94	8.00 ± 2.96	-3.020	0.004
TG [mmol/L, M (Q1, Q3)]	1.57(1.22,1.98)	2.95(1.62,4.24)	-2.715	0.007
HDL-C (mmol/L,‾x ± s)	1.34 ± 0.38	1.47 ± 0.46	-1.140	0.259
LDL-C [mmol/L, M (Q1, Q3)]	2.97(2.42,4.40)	3.90(3.04,6.35)	-1.902	0.057
Cys-C [mg/L, M(Q1,Q3)]	1.11(0.89,1.74)	0.83(0.69,0.92)	-3.944	< 0.001
TG/Cys-C ratio [M(Q1,Q3)]	1.24(0.75,1.91)	2.78(1.82,5.01)	-4.126	< 0.001
Scr [umol/L, M(Q1,Q3)]	118.00(73.75,145.50)	64.00(47.00,82.00)	-3.399	0.001
UA (umol/L, ‾x ± s)	319.57 ± 75.78	346.39 ± 84.90	-1.254	0.215
eGFR(ml/min /1.73m2, x ± s)	72.30 ± 36.59	104.20 ± 40.54	-3.110	0.003
UPE, [g/24 h, M(Q1,Q3)]	5.04(3.89,8.00)	3.83(2.14,7.47)	-0.895	0.371
Hematuria(number, %)	16(64)	19(56)	0.600	0.438
FIB (g/L,‾x ± s)	4.34 ± 1.52	4.27 ± 1.13	0.199	0.843

*DR* diabetic retinopathy, *BMI* body mass index, *SBP* systolic blood pressure, *DBP* diastolic blood pressure, *HbA1c* hemoglobin A1c, *Hb* hemoglobin, *FPG* fasting blood-glucose, *ALB* albumin, *TC* total cholesterol, *TG* triglyceride, *HDL-C* high-density lipoprotein Cholesterol, *LDL-C* low-density lipoprotein cholesterol, *Cys-C* Cystatin C, *TG/Cys-C ratio* Triglyceride / cystatin C ratio, *Scr* Serum creatinine, *UA* uric acid, *eGFR* estimated glomerular filtration rate, *UPE* urinary protein excretion obtained over 24 h, *FIB* Fibrinogen

### Analysis of DKD related factors

Logistic regression analysis showed that TG/Cys-C ratio (OR = 0.429) was a protective factor for DKD in patients with type 2 diabetes and proteinuria. Diabetic retinopathy (OR = 7.054) and systolic blood pressure (OR = 1.041) were risk factors for DKD in patients with type 2 diabetes and proteinuria (*P* < 0.05) (Table 2).

**Table 2:** Results of multivariate logistic regression analysis of DKD

Clinical parameters	B	S.E	Wald ^2^	P	OR (95% CI)
TG/Cys-C ratio	-0.845	0.323	6.865	0.009	0.429(0.228,0.808)
DR	-4.250	0.847	5.315	0.021	7.054(1.340,37.133)
SBP, mmHg	-0.171	0.020	3.938	0.047	1.041(1.000,1.082)

*SBP* systolic blood pressure, *DR* diabetic retinopathy, *TG/Cys-C ratio* Triglyceride / cystatin C ratio

### ROC curve analysis

The area under the curve of TG/Cys-C ratio, diabetic retinopathy and systolic blood pressure for DKD prediction was 0.816, 0.721 and 0.704, respectively. The sensitivity was 84%, 56% and 76%, respectively; The specificity was 67.6%, 88.2% and 52.9% respectively (*P* < 0.05) (Table 3).

**Table 3:** ROC curves for predicting diabetic kidney disease

Clinical parameters	AUC	95%CI	P	Youden	threshold	sensitivity(%)	specificity(%)
TG/Cys-C ratio	0.816	0.711 ~ 0.922	< 0.001	0.516	2.43	84	67.6
DR	0.721	0.583 ~ 0.860	0.004	0.442	-	56	88.2
SBP, mmHg	0.704	0.567 ~ 0.840	0.008	0.289	139	76	52.9

*SBP* systolic blood pressure, *DR* diabetic retinopathy, *TG/Cys-C ratio* Triglyceride / cystatin C ratio

### Patient characteristics in a prospective study

There was no significant difference in age, gender, BMI, smoking history, drinking history, diabetic retinopathy history and diabetes course between the two groups. There were no significant differences in HbA1C, FPG, ALB, TC, HDL-C, LDL-C, uric acid, urine protein, hematuria and fibrinogen between 2 groups (*P* > 0.05). Compared with TG/ Cys-C ≥ 2.43 group, TG/ Cys-C < 2.43 group had a higher proportion of hypertension, and the most obvious increase in systolic blood pressure, lower hemoglobin level, higher serum creatinine level, and lower glomerular filtration rate (Table 4).

**Table 4:** Comparison of clinical characteristics between the two groups

Clinical parameters	TG/Cys-C ratio < 2.43 Group(*n* = 29)	TG/Cys-C ratio ≥ 2.43 Group(*n* = 8)	Z/t/x^2^	*P* value
Male(number, %)	20(70)	6(75)	0.109	0.741
Age (years,‾x ± s)	50.00 ± 11.33	53.63 ± 10.25	0.816	0.420
Hypertension(number, %)	26(89.66)	3(37.5)	10.065	0.002
Tobacco smoking(number, %)	6(20.69)	1(12.5)	0.274	0.601
Alcohol drinking(number, %)	5(17.24)	1(12.5)	0.104	0.747
DR(number, %)	14(48.28)	1(12.5)	3.329	0.068
BMI (kg/m2, ‾x ± s)	25.13 ± 3.64	25.35 ± 3.63	0.154	0.879
SBP(mmHg, ‾x ± s)	155.66 ± 19.89	135.00 ± 17.12	-2.670	0.011
DBP (mmHg, ‾x ± s)	91.14 ± 11.38	82.88 ± 10.56	-1.844	0.074
Diabetes duration[month, M (Q1, Q3)]	66(24,120)	12(1,42)	-1.439	0.150
HbA1C [%, M (Q1, Q3)]	7.95(6.60,10.70)	7.30(6.85,7.70)	-0.343	0.732
Hb [g/L, M (Q1, Q3)]	116(99,133)	153(137,157)	-2.823	0.005
FPG [mmol/L, M (Q1, Q3)]	7.00(5.95,9.71)	7.36(6.71,7.66)	-0.664	0.507
ALB(g/L,‾x ± s)	31.93 ± 8.89	29.59 ± 6.93	-0.688	0.496
TC (mmol/L,‾x ± s)	6.42 ± 2.02	7.23 ± 1.46	1.052	0.300
HDL-C [mmol/L, M (Q1, Q3)]	1.13(0.83,1.44)	1.10(0.98,1.12)	-0.572	0.56
LDL-C (mmol/L,‾x ± s)	4.32 ± 1.67	4.54 ± 1.58	0.332	0.742
Scr [umol/L, M(Q1,Q3)]	88(70,117)	75(67,76.5)	-2.492	0.013
UA (umol/L, ‾x ± s)	371.17 ± 78.46	328.13 ± 93.77	-1.319	0.196
eGFR(ml/min /1.73m2, x ± s)	66.86 ± 31.94	95.78 ± 17.69	2.443	0.020
UPE, [g/24 h, M(Q1,Q3)]	3.59(2.42,7.60)	4.05(3.27,4.99)	-0.405	0.686
Hematuria(number, %)	15(51.72)	2(25)	1.803	0.179
FIB (g/L,‾x ± s)	4.57 ± 1.16	4.10 ± 0.99	-1.033	0.309

*DR* diabetic retinopathy, *BMI* body mass index, *SBP* systolic blood pressure, *DBP* diastolic blood pressure, *HbA1c* hemoglobin A1c, *Hb* hemoglobin, *FPG* fasting blood-glucose, *ALB* albumin, *TC* total cholesterol, *HDL-C* high-density lipoprotein Cholesterol, *LDL-C* low-density lipoprotein cholesterol, *Scr* Serum creatinine, *UA* uric acid, *eGFR* estimated glomerular filtration rate, *UPE* urinary protein excretion obtained over 24 h, *FIB* Fibrinogen

### Comparison of DKD positive detection rates between the two groups

Among 37 patients with type 2 diabetes mellitus complicated with proteinuria, 29 patients had TG/Cys-C ratio < 2.43, and 8 patients had TG/Cys-C ratio ≥ 2.43. Of the 29 patients, 22 were diagnosed as DKD and 7 were diagnosed as NDKD. Among the 8 cases, 2 cases were diagnosed as DKD, 6 cases were diagnosed as NDKD by pathology. The sensitivity was 91.67%, specificity was 46.15%, positive predictive value was 75.80%, negative predictive value was 75% (Table 5).

**Table 5:** Comparison of DKD positive detection rates between the two groups

Study Group	DKD	NDKD
TG/Cys-C ratio < 2.43(number)(*n* = 29)	22	7
TG/Cys-C ratio ≥ 2.43(number)(*n* = 8)	2	6
Statistical	value	
*P* value	0.013	
sensitivity	91.67%	
specificity	46.15%	
positive predictive value	75.8%	
negative predictive value	75%	

## Discussion

The pathological changes of DKD are complex, many diseases are hidden, showing chronic progressive development, and eventually progress to End-stage renal disease (ESRD), which seriously affects the quality of life and life safety of patients. At present, DKD is the main cause of chronic kidney disease and ESRD in the world [11]. timely prevention and diagnosis of DKD is an important part of the treatment of diabetic patients. Up to now, a large number of researchers have explored the early predictive markers of DKD, including proteomics and genomics. However, these new biomarkers are still lack of stability, and the detection cost is high, which is difficult to promote in clinical practice [12]. Therefore, this study relies on the pathological diagnosis of renal biopsy as the gold standard, and explores the convenient and sensitive non-invasive indicators that can predict the diagnosis of DKD.

In this review, we retrospectively reviewed the recent diagnosis of type 2 diabetes mellitus complicated with proteinuria in our city. According to the diagnostic criteria of renal biopsy, 25 cases were diagnosed as DKD and 34 cases diagnosed as NDKD. There are many pathological types in NDKD group, among which membranous nephropathy is the most common pathological type in NDKD group, which is similar to the results reported in other Asian Studies [3, 13].

By reviewing and comparing the clinical indexes of the two groups, it was found that the TG/Cys-C ratio was significantly different in DKD and NDKD patients. TG/Cys-C also considered the status of dyslipidemia and indicators of renal dysfunction in T2DM patients with renal impairment. At present, there is no definite conclusion about the role of TG/Cys-C in differentiating DKD and NDKD. Studies have shown that hyperlipidemia is an independent risk factor for the progression of DKD [14]. In vivo and in vitro experiments have shown that hyperlipidemia can cause glomerulosclerosis and tubulointerstitial sclerosis by stimulating resident renal cells to produce fibrotic cytokines and chemokines, leading to infiltration of monocytes or macrophages into glomerular tissue to promote extracellular matrix deposition in tubulointerstitial and mesangial cells [15]. Animal experiments have shown that lipid accumulation, lipid toxicity and lipid metabolism disorders are related to diabetic kidney damage [16]. In addition, T2DM patients with other kidney diseases, especially primary nephrotic syndrome, are closely associated with hyperlipidemia. Studies [17] have found that dyslipidemia not only promotes glomerulosclerosis and tubulointerstitial damage, but also induces or aggravates renal and external vascular lesions, leading to thromboembolism and other serious complications. Serum Cys-C, a low molecular weight protein, is an endogenous cysteine protease inhibitor and is not affected by inflammation, muscle content, gender, age and other factors [18]. Serum cystatin C is an ideal indicator of glomerular filtration rate [19]. Previous studies [7] have shown that serum Cys-C is an early sensitive biomarker in DKD patients. Therefore, this study intends to explore the clinical significance of TG/ Cys-C in the differential diagnosis of DKD and NDKD.

Previous meta-analysis [20] showed that TG levels were higher in NDKD patients than in DKD patients, and the specific mechanism remains to be further studied. In the univariate regression analysis, the TG level in the DKD group was lower than that in the NDKD group, and the Cys-C level in the DKD group was higher than that in the NDKD group. Here, we used the ratio of these two indexes for analysis. When the TG/Cys-C ratio was included in the regression, the results showed that the TG/Cys-C ratio was significantly better than the two indexes alone in distinguishing DKD and NDKD. The multivariate regression analysis showed that the TG/Cys-C ratio was an independent protective factor for DKD in T2DM patients with proteinuria.

This retrospective study shows that in patients with type 2 diabetes and proteinuria, DKD patients have higher blood pressure and creatinine levels, lower glomerular filtration rate and hemoglobin level. This indicates that the level of renal function in patients with DKD is relatively poor in the course of diabetes mellitus. Besides the combined effect of renal hemodynamic disorder, the sensitivity of insulin receptor is reduced, the function of islet is reduced, and the time of exposure to high glucose environment is prolonged. This leads to the increased risk of DKD in patients [21]. All the above confounding factors were included in the multivariate logistic regression analysis. The results showed that TG/Cys-C ratio was a protective factor for DKD in patients with type 2 diabetes and proteinuria, that is, the TG/Cys-C ratio increased by one unit, and the risk of DKD decreased by 0.571 times. Diabetic retinopathy and systolic blood pressure are independent risk factors for DKD in patients with type 2 diabetes and proteinuria. The risk of DKD in patients with diabetic retinopathy is 6.054 times higher than that in patients without diabetic retinopathy. When systolic blood pressure increased by one unit, the risk of DKD increased by 0.041 times. The results of this retrospective study show that the lower the TG/Cys-C ratio is, the greater the likelihood of diagnosing DKD in type 2 diabetic patients with proteinuria. Conversely, it is possible to alert NDKD lesions to renal biopsy indications.

It is generally believed that the duration of diabetes, the absence of diabetic retinopathy, hematuria or unexplained acute kidney injury may be a useful indicator of renal biopsy in patients with type 2 diabetes combined with dominant proteinuria [22]. Previous studies have found that duration of diabetes was the strongest predictor of whether NDKD or DKD was identified on biopsy [23]. However, other studies confirm that NDKD can not be completely excluded in patients with type 2 diabetes mellitus with longer duration of diabetes and diabetic retinopathy [24]. In addition, some studies have suggested that the presence of microhaematuria is an independent risk factor for NDKD [25]. However, the comparative analysis of DKD and NDKD patients in this retrospective study showed no significant difference in microhaematuria, which may be related to the small sample size of this study.

In order to evaluate the diagnostic value of TG/Cys-C ratio for DKD in patients with type 2 diabetes mellitus and dominant proteinuria, the ROC curve was constructed in this retrospective study. The results showed that the area under the DKD curve predicted by the TG/Cys-C ratio was 0.816, corresponding sensitivity and specificity were 84% and 67.6%, respectively, and the cut-off point value was 2.43. The results of this study showed that when the TG/Cys-C ratio was lower than 2.43 in patients with type 2 diabetes and proteinuria, it was suggested that the possibility of diagnosing DKD was too large. When the TG/Cys-C ratio was higher than 2.43, we should be alert to the possibility of combining NDKD. Further renal biopsy was recommended to make clear diagnosis and guide treatment.

In order to further verify the predictive value of TG/Cys-C ratio for the diagnosis of DKD, based on the results of previous retrospective studies, a prospective study was carried out in newly enrolled type 2 diabetic patients with proteinuria by taking the cut-off point value TG/Cys-C of the ratio as the defined value. The results showed that among the 37 patients with type 2 diabetes mellitus combined with proteinuria, TG/ Cys-C < 2.43 in 29 patients and TG/ Cys-C ≥ 2.43 in the other 8 patients. We analyzed the clinical characteristics of patients in the two groups, and the renal function level of patients in the TG/ Cys-C < 2.43 group was worse. We analyzed the pathological characteristics of renal biopsy in patients with type 2 diabetes. (a) DKD, defined by the presence of suggestive glomerular lesions like nodular sclerosis, diffuse mesangial sclerosis, mesangial expansion, basement membrane thickening, arteriolar hyalinosis, micro aneurysms and exudative lesions [26]. (b) NDKD, defined by any histological alteration different from the above-mentioned and suggestive of other renal diseases. There were 11 cases of membranous nephropathy and 2 cases of mesangial proliferative nephropathy. Among the 29 patients, 22 were diagnosed as DKD by renal pathology, and 6 of the 8 patients were diagnosed as NDKD by renal pathology. The sensitivity was 91.67%, the specificity was 46.15%, the positive predictive value was 75.80%, and the negative predictive value was 75.00%. In the population with TG/Cys-C ratio < 2.43, the proportion of pathological diagnosis of DKD by fine needle renal biopsy was significantly higher, which confirmed that TG / Cys-C ratio had a certain predictive value for the diagnosis of DKD.

In summary, the low level of TG/Cys-C is a protective factor for DKD in type 2 diabetic patients with proteinuria. TG/Cys-C ratio has important clinical guiding significance for the prediction and diagnosis of DKD.

This study has some limitations. Firstly, information bias cannot be avoided in this retrospective study. All patients' medical history data are from the internal electronic medical record system of the hospital, and there may be poor accuracy of medical history records. In addition, blood pressure measurement is greatly affected by emotion or activity, which is easy to cause blood pressure fluctuation and data deviation. Secondly, due to the limitation of clinical sample size, the cases collected in this study are only within the scope of this city and can not represent the population in all regions of the country, which limits the universal applicability of the research results to other groups. Finally, the occurrence and development process of DKD and NDKD diseases is extremely complex. There may be other influencing factors that have not been explored in the research, and more in-depth exploration is needed in the future.

## Data Availability

The datasets generated and/or analysed during the current study are not publicly available due [“It involves more privacy of patients”] but are available from the corresponding author on reasonable request.

## Declarations

### Competing of interest

The authors declare that they have no confict of interest.

## References

[1] Complications M, Care F (2021). Standards of Medical Care in Diabetes-2021. Diabetes Care.

[2] Arora P, Roychaudhury A, Pandey R (2020). Non-diabetic Renal Diseases in Patients with Diabetes Mellitus Clinicopathological Correlation. Indian J Nephrol.

[3] Wang J, Han Q, Zhao L (2019). Identification of clinical predictors of diabetic nephropathy and non-diabetic renal disease in Chinese patients with type 2 diabetes, with reference to disease course and outcome. Acta Diabetol.

[4] Liu D, Huang T, Chen N (2018). The modern spectrum of biopsy-proven renal disease in Chinese diabetic patients-a retrospective descriptive study. PeerJ.

[5] Yang YZ, Wang JW, Wang F (2017). Incidence, Development, and Prognosis of Diabetic Kidney Disease in China: Design and Methods. Chin Med J (Engl).

[6] Song KH, Jeong JS, Kim MK (2019). Discordance in risk factors for the progression of diabetic retinopathy and diabetic nephropathy in patients with type 2 diabetes mellitus. J Diabetes Investig.

[7] Elsayed MS, El Badawy A, Ahmed A, Omar R, Mohamed A (2019). Serum cystatin C as an indicator for early detection of diabetic nephropathy in type 2 diabetes mellitus. Diabetes Metab Syndr.

[8] Diagnosis and classification of diabetes mellitus. Diabetes Care. 2013. 36 Suppl 1(Suppl 1): S67–74. 10.2337/dc13-S067 PMC3537273 23264425

[9] Classification and Diagnosis of Diabetes (2022). Standards of Medical Care in Diabetes-2022. Diabetes Care.

[10] Levey AS, Coresh J, Greene T (2007). Expressing the Modification of Diet in Renal Disease Study equation for estimating glomerular filtration rate with standardized serum creatinine values. Clin Chem.

[11] Zhu H, Liu M, Yu H (2017). Glycopatterns of Urinary Protein as New Potential Diagnosis Indicators for Diabetic Nephropathy. J Diabetes Res.

[12] Jiang W, Wang J, Shen X (2020). Establishment and Validation of a Risk Prediction Model for Early Diabetic Kidney Disease Based on a Systematic Review and Meta-Analysis of 20 Cohorts. Diabetes Care.

[13] Dong Z, Wang Y, Qiu Q (2016). Clinical predictors differentiating non-diabetic renal diseases from diabetic nephropathy in a large population of type 2 diabetes patients. Diabetes Res Clin Pract.

[14] Liu S, Guo Q, Han H (2016). Clinicopathological characteristics of non-diabetic renal disease in patients with type 2 diabetes mellitus in a northeastern Chinese medical center: a retrospective analysis of 273 cases. Int Urol Nephrol.

[15] Almquist T, Jacobson SH, Mobarrez F, Näsman P, Hjemdahl P (2014). Lipid-lowering treatment and inflammatory mediators in diabetes and chronic kidney disease. Eur J Clin Invest.

[16] Su K, Yi B, Yao BQ (2020). Liraglutide attenuates renal tubular ectopic lipid deposition in rats with diabetic nephropathy by inhibiting lipid synthesis and promoting lipolysis. Pharmacol Res.

[17] Agrawal S, Zaritsky JJ, Fornoni A, Smoyer WE (2018). Dyslipidaemia in nephrotic syndrome: mechanisms and treatment. Nat Rev Nephrol.

[18] Akpınar K, Aslan D, Fenkçi SM (2021). Assessment of estimated glomerular filtration rate based on cystatin C in diabetic nephropathy. J Bras Nefrol.

[19] Arceo ES, Dizon GA, Tiongco R (2019). Serum cystatin C as an early marker of nephropathy among type 2 diabetics: A meta-analysis. Diabetes Metab Syndr.

[20] Liang S, Zhang XG, Cai GY (2013). Identifying parameters to distinguish non-diabetic renal diseases from diabetic nephropathy in patients with type 2 diabetes mellitus: a meta-analysis. PLoS ONE.

[21] Huang Y, Jin L, Yu H (2020). SNPs in PRKCA-HIF1A-GLUT1 are associated with diabetic kidney disease in a Chinese Han population with type 2 diabetes. Eur J Clin Invest.

[22] Fiorentino M, Bolignano D, Tesar V (2017). Renal biopsy in patients with diabetes: a pooled meta-analysis of 48 studies. Nephrol Dial Transplant.

[23] Sharma SG, Bomback AS, Radhakrishnan J (2013). The modern spectrum of renal biopsy findings in patients with diabetes. Clin J Am Soc Nephrol.

[24] Lin YL, Peng SJ, Ferng SH, Tzen CY, Yang CS (2009). Clinical indicators which necessitate renal biopsy in type 2 diabetes mellitus patients with renal disease. Int J Clin Pract.

[25] Bermejo S, González E, López-Revuelta K (2020). Risk factors for non-diabetic renal disease in diabetic patients. Clin Kidney J.

[26] Tervaert TW, Mooyaart AL, Amann K (2010). Pathologic classification of diabetic nephropathy. J Am Soc Nephrol.

